# Why does the m6A writer complex require so many proteins?

**DOI:** 10.1371/journal.pbio.3003386

**Published:** 2025-09-15

**Authors:** Kiran Pandey, Hui Xian Poh, Samie R. Jaffrey

**Affiliations:** Department of Pharmacology, Weill Cornell Medicine, Cornell University, New York, New York, United States of America

## Abstract

The conversion of adenosine to *N*^6^-methyladenosine (m6A) is the most common internal modification made to eukaryotic mRNA and is involved in post-transcriptional control of gene expression. When dysregulated, m6A has been linked to a variety of diseases, including cancer. Unlike other RNA methyltransferases, m6A conversion is catalyzed by a multi-protein writer complex with only one catalytic subunit. So why is the m6A writer complex composed of so many proteins? This Unsolved Mystery explores the possible functions of the proteins of the m6A writer complex and discusses why it might require multiple subunits. Understanding the roles of these proteins could provide insight into how m6A is regulated and reveal new strategies for targeting diseases linked to m6A dysregulation.

## Abstract 2

Post-transcriptional gene regulation through m6A deposition is dysregulated in many diseases and is catalyzed by a multi-protein writer complex. This Unsolved Mystery investigates why the writer complex requires multiple subunits and what roles its individual proteins might have in m6A regulation.

## Introduction

*N*^6^-methyladenosine (m6A) is the most abundant internal mRNA base modification in eukaryotes and is pivotal in the regulation of post-transcriptional gene expression. This modification impacts several aspects of RNA metabolism, primarily mRNA stability, but also splicing, translation efficiency and subcellular localization [1]. m6A modification is targeted to mRNAs in a highly selective manner, with mRNAs encoded by genes containing large internal exons being particular targets for methylation, and levels of m6A are tightly regulated in response to both cellular and environmental cues, resulting in corresponding changes in gene expression [2]. m6A-enriched mRNAs encode proteins that have roles in numerous biological processes, particularly cell differentiation and stress responses; consequently, dysregulation of the m6A pathway is implicated in a variety of pathological states, including cancer, in which altered m6A levels influence the expression of oncogenes and tumor suppressors [3].

**Fig 1:**
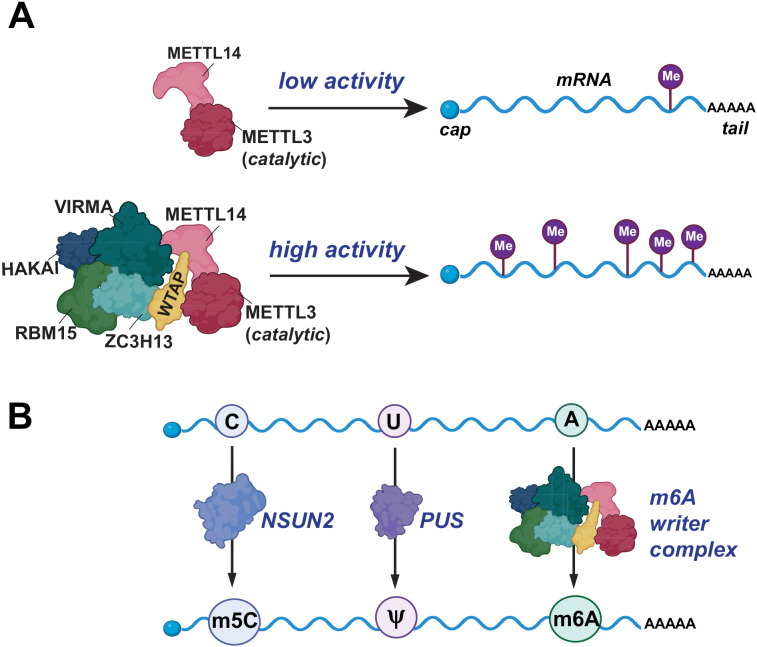
m6A methylation requires a multi-protein writer complex. The m6A writer complex is a multi-component assembly that deposits *N*^6^-methyladenosine (m6A) on mRNA in a transcriptome-wide yet site-selective manner. In the writer complex, only METTL3 contains the catalytic domain, but optimal methylation requires additional non-catalytic auxiliary proteins. Unlike other RNA modifications that are catalyzed by single enzymes, m6A methylation requires a multi-protein complex. By contrast, NSUN2 (a cytosine-5 RNA methyltransferase) and pseudouridine synthase (PUS) enzymes typically function as single-protein modifiers that act on defined RNA substrates. NSUN2 catalyzes 5-methylcytosine (m5C) deposition primarily on tRNAs, but also on vault RNAs, 7SK RNA and some mRNAs, where it regulates the RNA’s stability and translation. PUS enzymes convert uridine to pseudouridine (Ψ) on tRNAs, rRNAs, snRNAs and a subset of mRNAs in a site-specific manner. The necessity for a multi-protein complex for m6A deposition and the roles of each writer complex component remains a mystery. Created in BioRender. Jaffrey, S. (2025) https://BioRender.com/hffsuxx.

The deposition of m6A is mediated by a multi-protein complex, commonly referred to as the m6A writer complex or methyltransferase complex, consisting of seven proteins, only one of which is catalytically active (Fig 1) [4]. Compared to other RNA methyltransferases, the mammalian m6A writer complex is unusually complex. For example, METTL16, which catalyzes m6A modifications on U6 spliceosomal RNA, does not require any auxiliary proteins for its catalytic activity. Although METTL16 associates with proteins such as La, LARP7 and MEPCE [5], these proteins are not thought to be required for METTL16-mediated m6A formation. Similarly, METTL5, which modifies 18S rRNA, forms a simple heterodimer with the TRMT112 co-factor [6]. Even other RNA modifications, such as 5-methylcytosine (m5C) and pseudouridine in mRNA and tRNA are catalyzed by single enzymes rather than by complexes (Fig 1) [7]. Thus, the m6A writer complex with its multiple component proteins is unusual, and we have limited understanding of the roles played by its non-catalytic subunits.

In this Unsolved Mystery, we provide an overview of what is currently known about the proteins of the m6A writer complex and expand on possible functions of the auxiliary subunits and how they may contribute to regulatory functions such as modulating substrate specificity, organizing the spatial localization of the complex, or coordinating with other RNA processing pathways. Understanding these mechanisms will be essential for elucidating how the m6A landscape adapts to various physiological stimuli and how its dysregulation contributes to disease. Furthermore, a deeper understanding of the functional roles of the auxiliary proteins in the m6A writer complex may provide new opportunities for therapeutic interventions targeting m6A dysregulation in disease contexts.

## Discovery of the m6A writer complex

The m6A writer complex was discovered in the 1990s through the work of Bokar and Rottman [8]. Using biochemical fractionation techniques, including gel filtration and glycerol gradient sedimentation, Bokar and colleagues successfully characterized mRNA methyltransferase activity from HeLa cell nuclear extracts. The methyltransferase activity was observed by combining two fractions from a sizing column that were predicted to contain an approximately 875 kDa complex and an approximately 200 kDa complex. The 200-kDa fraction contained the 70 kDa catalytic subunit that was identified by its ability to crosslink to radioactive *S*-adenosylmethionine, the methyl donor for m6A formation [8]. This catalytic subunit, containing 580 amino acids, was termed MT-A70 and is now known as N(6)-adenosine-methyltransferase catalytic subunit METTL3 (hereafter METTL3).

Further advances occurred in 1997 when Bokar and Rottman cloned and purified METTL3, confirming its role as a key catalytic component of the m6A writer complex [9]. Despite this breakthrough, METTL3 alone could not recapitulate the full methylation activity observed in nuclear extracts [8], highlighting the need for auxiliary proteins to act together with METTL3 for optimal m6A methylation (Fig 1). These seminal studies established that m6A methylation is catalyzed by a multi-protein complex rather than by METTL3 alone.

Subsequent work identified the remaining proteins of the writer complex. Wilms-tumor associated protein (WTAP) was the first to be discovered through plant genetic studies as an interactor of MTA, the plant homolog of METTL3 [10]. Although this study demonstrated that the WTAP plant homolog FIP37 binds MTA, it did not establish whether WTAP is required for m6A modification. A clearer role emerged from yeast studies, in which the WTAP homolog MUM2 interacted with the METTL3 homolog IME4 and was required for mRNA methylation [11]. Later, a study of m6A in mammalian cells showed that WTAP (44 kDa, 396 amino acids), is required for m6A formation and facilitates the localization of METTL3 to nuclear compartments [4]. This and two other studies also identified METTL14 (52 kDa, 456 amino acids) as a METTL3-associated protein [4,12,13]. METTL14 has a domain with sequence similarity to canonical RNA methyltransferases. Although one study originally proposed METTL14 as a separate m6A-forming enzyme [13], it is now known that METTL14 is a catalytically inactive protein that binds and allosterically activates METTL3 to form the functional core of the writer complex [14–16].

An early proteomic study of WTAP-binding proteins [17] further revealed the complexity of the m6A writer complex. Although this study was not focused on the function of WTAP in the m6A writer complex, it revealed that WTAP binds METTL3 and METTL14, as well as the other main writer complex members vir-like m6A methyltransferase associated (VIRMA, also known as KIAA1429; 200 kDa, 1812 amino acids), zinc finger CCCH domain-containing protein 13 (ZC3H13; 196 kDa, 1668 amino acids), RNA-binding protein 15 (RBM15; 107 kDa, 977 amino acids) and Cbl proto-oncogene like 1 (CBL, also known as HAKAI; 55 kDa, 491 amino acids). VIRMA was shown to be a functional member of the writer complex in a study of METTL3-interacting proteins in mammalian cells [18] and in Drosophila studies of sex-specific splicing [19]. In both cases, VIRMA interacted with METTL3 and was required for m6A deposition. ZC3H13 and its Drosophila counterpart Flacc, were identified as novel interactors of the writer machinery through interactome analyses using RBM15 (mouse) and Nito (fly) as bait [20]. RBM15 was discovered in studies of the noncoding RNA XIST in mammals [21] and in Drosophila [19], and shown in both cases to be part of the m6A writer complex and necessary for transcriptome-wide deposition of m6A. HAKAI was originally characterized as a RING-type E3 ubiquitin ligase in epithelial-mesenchymal transition pathways in epithelial cells [22], and was found to be a conserved member of the writer complex based on its co-purification with WTAP and its homolog in vertebrates and plants [17,20,23]. Genetic studies in both Arabidopsis and Drosophila showed that HAKAI is required for stabilizing the writer complex components and for maintaining global m6A levels [24,25].

## What is known about the function of the auxiliary proteins?

Although METTL3 and METTL14 form the core enzymatic machinery of the m6A writer complex, the necessity of auxiliary proteins raises questions about their functional roles. In the following section, we examine how these components might affect methylation by influencing the targeting, localization and dynamic regulation of the m6A writer complex.

### Targeting METTL3-METTL14 to transcription-associated domains

The auxiliary subunits of the m6A writer complex are needed for methylation, suggesting that they either activate METTL3-METTL14 or position METTL3-METTL14 near their targets. However, current models for m6A formation could be better explained by a single enzyme than by a multi-protein complex. The idea is that any DRACH site (D = A/G/U, R = A/G, A = m6A site, C = C, H = A/C/U) [26] will be methylated if it is accessible to the METTL3-METTL14 heterodimer, with specificity for the DRACH site coming from the RNA-binding domain of METTL14 [14–16,27]. However, if a DRACH site is occluded by the exon junction complex (EJC), a multi-protein complex that is bound to splice sites, then the DRACH site will not be methylated. This model of EJC blockade at DRACH sites explains why m6A is not generally found around splice junctions but instead is present in the central region of long exons, which are distant from the EJC [28–30]. In this model of ‘suppressive’ methylation, METTL3-METTL14 alone could, in theory, explain the observed methylation pattern, given that METTL3 simply methylates any DRACH sites that it encounters (i.e., DRACH sites that are not occluded by the EJC). The auxiliary proteins in this model may help to position the catalytic core, modulate RNA structure or influence accessibility indirectly. Furthermore, given the large size and dynamic assembly of both the EJC and the m6A writer complex, interactions between these complexes cannot be ruled out and may function to further refine methylation selectivity. Thus, the mechanism of RNA methylation likely involves additional mechanisms that utilize the auxiliary proteins.

A problem with the suppressive methylation model comes from evidence that m6A deposition occurs co-transcriptionally, rather than post-transcriptionally (or at least post-splicing), as the EJC-mediated suppressive model implies. Initial studies showed that m6A methylation occurs across approximately the same timescale as mRNA capping [31], which is known to happen almost immediately after transcription [32,33]. A study measuring m6A levels in chromatin-associated nascent RNA and nuclear RNA showed that m6A levels are highest in the chromatin-associated fraction [34,35], suggesting that they are deposited shortly after transcription. Mapping studies also showed high levels of m6A in newly transcribed mRNA [36,37]. Crucially, in these studies, m6A was found in introns of the nascent transcriptomes, albeit at low amounts [28,34,36,37], indicating that the process of m6A methylation begins before introns are spliced out, and thus before the EJC has assembled. Furthermore, m6A has a role in regulating splicing [36–38], which could only occur if m6A deposition occurred before splicing. In Drosophila, METTL3 has been observed on polytene chromosomes, supporting the hypothesis that the m6A methylation process is closely linked to transcription [39].

These prior studies place m6A methylation as an early event in transcription, which might seem to contradict the suppressive model; however, it is possible that this methylation occurs both co-transcriptionally and post-splicing. Indeed, a recent study has shown that nuclear retention of mRNA leads to increased m6A methylation [40], suggesting that there is some continuous methylation that occurs over time, even after the initial transcription step. Co-transcriptional m6A methylation can help to explain why the complex requires auxiliary proteins: if methylation is coordinated with splicing or other processing events, the m6A writer complex may need to interact with splicing factors, RNA polymerase II (RNA Pol II) and/or other components of the transcription machinery. Some evidence for these interactions has been reported. Notably, the RNA Pol II B subunit was detected in a proteomic dataset showing proteins interacting with VIRMA, but not in similar datasets for proteins interacting with METTL3 or WTAP [41]. This raises the possibility that VIRMA is physically closer to RNA Pol II than METTL3 or WTAP. However, further studies are needed to validate any functional interactions between VIRMA and/or other m6A writer complex proteins and RNA Pol II.

Another function of the auxiliary proteins may be to influence RNA Pol II kinetics; indeed, a study found that slower transcription rates enhanced m6A deposition on mRNAs [42]. Another function of m6A during transcription may be to regulate promoter-proximal pausing of RNA Pol II, a key checkpoint after transcriptional initiation [43]. m6A modification of the nascent RNA releases RNA Pol II from its paused state, leading to transcript elongation [44,45]. Loss of METTL3 catalytic function, and thereby m6A deposition, makes paused RNA Pol II more susceptible to integrator-mediated cleavage, resulting in premature termination of transcription. The auxiliary proteins may therefore control rates of transcription and thus methylation stoichiometry, or may facilitate methylation of nascent RNA; however, these functions would not explain the essential role of auxiliary proteins in m6A formation.

Other forms of ‘active recruitment’ may occur, including specific recruitment of the m6A writer complex to chromatin or nascent RNA, again potentially mediated by auxiliary protein(s). METTL14 and/or RBM15 might have these functions. RBM15 has been proposed to interact with proteins linked to splicing, such as Aly/REF [46] and PNMT1 [47], suggesting that it might anchor the m6A writer complex to nascent RNA in the early stages of splicing, whereas METTL14 has been proposed to bind to chromatin regions marked by H3K36me3 and thus allow the m6A writer complex to methylate nascent RNAs as they are being transcribed [35].

Moreover, the m6A writer complex methylates not just mRNAs but also various chromatin-associated regulatory RNAs (carRNAs), such as promoter-associated RNAs (paRNAs), enhancer RNAs (eRNAs) and transcripts derived from transposable elements [36,45,48]. Recruitment of the m6A writer complex to promoters and enhancers can lead to methylation of these RNAs, potentially post-transcriptionally. Interestingly, some evidence suggests that m6A writer complex localization to chromatin can promote nascent RNA synthesis, and loss of METTL3 leads to reduced transcriptional output at these sites [45].

It remains to be investigated what proportion of m6A deposition is shaped by suppressive mechanisms mediated by the EJC versus active recruitment by auxiliary proteins, chromatin marks or transcriptional regulators. These mechanisms are not mutually exclusive and may operate in parallel, with their relative contributions varying across transcripts, cell types and species.

### Targeting METTL3–METTL14 to specific nuclear domains

In addition to transcription-associated localization, auxiliary proteins may guide the m6A writer complex to specific nuclear compartments such as speckles. For methylation to occur, the m6A writer complex needs to be in the nucleus [1], as methylation occurs during or shortly after transcription [49]. Thus, auxiliary proteins may function to ensure nuclear localization and retention of the m6A writer complex. Indeed, depletion of WTAP leads to the relocalization of METTL3 into the cytosol and a subsequent loss of m6A in mRNA [4]. This relocalization occurs despite the presence of a nuclear localization signal (NLS) within METTL3 [50].

Recent studies have also emphasized the importance of nuclear speckles, specialized nuclear compartments involved in RNA processing [50,51], as important sites for the localization of the m6A writer complex. Although it is unclear if m6A formation occurs in nuclear speckles, the localization to speckles may be important for proper regulation of m6A biology. Notably, WTAP, ZC3H13 and other auxiliary proteins are known to localize to speckle-like structures in the nucleus [40,50], and depletion of these proteins leads to the mislocalization of METTL3 and loss of m6A. Conceivably, one auxiliary protein function could be to ensure localization of the m6A writer complex to the nucleus or specific speckle compartments.

Many nuclear domains are formed as a result of phase separation, such as Cajal bodies and nuclear speckles [52]. The m6A writer complex may be localized as a result of, or even activated by, phase separation. Several members of the m6A writer complex, including METTL3, METTL14 and WTAP, undergo phase separation, forming condensates, potentially within nuclear speckles [51]. In the case of METTL3, this may be driven by its association with the methyl donor S-adenosylmethionine (SAM). Condensates may provide additional targeting of the m6A writer complex, particularly at sites of active transcription. Therefore, the localization of the core complex to nuclear speckles and/or their phase separation may be key functions of auxiliary proteins, ensuring efficient and context-specific RNA methylation.

## What roles might each auxiliary protein have?

**Fig 2:**
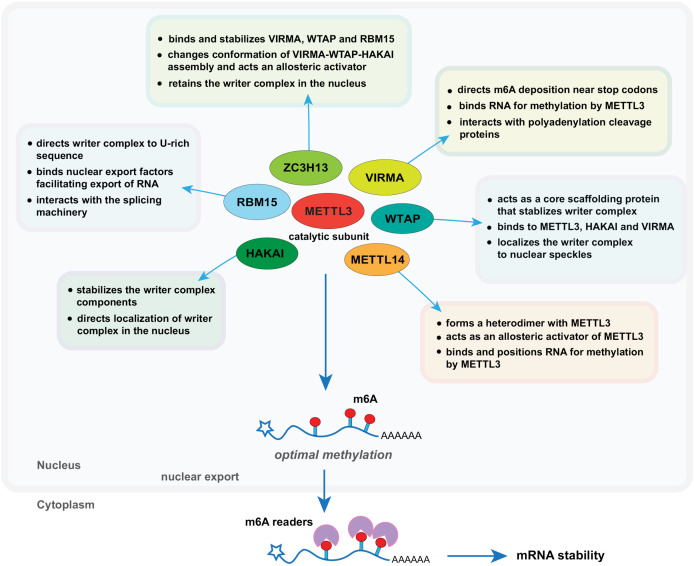
Potential functions of the components of the m6A writer complex. This schematic summarizes the proposed potential roles of METTL3, METTL14, WTAP, VIRMA, ZC3H13, RBM15/15B and HAKAI in the m6A writer complex. METTL3 serves as the catalytic subunit, while METTL14 stabilizes METTL3 and contributes to RNA binding. WTAP functions as a scaffold for nuclear localization and complex assembly. VIRMA is implicated in transcript selectivity and preferential 3′ UTR methylation. ZC3H13 anchors the complex to the nuclear matrix and may regulate its activity state. RBM15 is thought to act as adapter proteins that recruit the m6A writer complex to specific RNA substrates, particularly to U-rich regions of transcripts. HAKAI, a putative E3 ubiquitin ligase, may modulate complex stability or respond to cellular stress, although its exact function remains unclear. Together, these components coordinate methylation in a transcript- and context-specific manner.

In the previous section, we described the known functions of each auxiliary protein. In this section, we speculate on their possible roles in writer complex assembly and m6A deposition (Fig 2).

### METTL14: A core member of the heterodimeric methyltransferase

The role of METTL14 in the m6A writer complex was initially misunderstood, with early studies suggesting that it was a second methyltransferase that forms m6A [13]. However, structural studies conducted by several groups [14,16] have clarified that METTL14 is not an enzyme, but instead forms a heterodimer with METTL3, acting as an allosteric activator of METTL3. The structural studies and subsequent biochemical analysis [50,53] have revealed that METTL14 recognizes and optimally positions RNA for methylation by METTL3 bound to SAM. Early reports of METTL14 catalytic activity [13] were likely due to contamination with insect-derived METTL3 obtained from the cells used for METTL14 purification [14].

The stoichiometry between METTL3 and METTL14 is critical for efficient m6A deposition, as an imbalance in the levels of either subunit does not lead to increased methylation. For example, an elevation in METTL3 levels without a corresponding increase in METTL14 does not result in additional m6A marks [14]. METTL14 appears to stabilize METTL3, which would otherwise be unstable when not in a heterodimeric complex [27,54]. Thus, in addition to binding and positioning RNA for methylation, METTL14 may also be needed for m6A formation by stabilizing METTL3.

### WTAP: A scaffolding protein and potential regulator of localization

Disruption of WTAP expression leads to a complete loss of m6A methylation [4], indicating its essential role in the m6A writer complex’s stability and function. WTAP acts as a scaffold connecting several other components of the m6A writer complex. The WTAP N-terminal domain (1–200 aa) binds METTL3 [4], while other regions of WTAP directly bind HAKAI [17]. A cryo-electron microscopy (cryo-EM) structure of WTAP also shows direct interaction between WTAP and VIRMA, and between WTAP and METTL3 [55]. Thus, WTAP may be a core scaffolding protein within the writer complex.

Following WTAP depletion, METTL3 and METTL14 lose their association with nuclear speckles and become distributed throughout the cell, suggesting that WTAP, or one of its binding partners, binds a core nuclear speckle protein [4]. However, it is still unclear whether mRNAs are methylated in speckles or if WTAP’s role is to bind and store METTL3 in speckles. A recent study [56] highlighted the role of nuclear speckles in storing spliceosome components and enhancing mRNA splicing efficiency for genes that are located closer to these regions. Thus, WTAP may store METTL3 in nuclear speckles, ensuring a supply of METTL3 for efficient methylation and coordination with splicing.

WTAP also interacts with proteins beyond the canonical m6A writer complex proteins [17]. These include the arginine/serine-rich domain-containing splicing proteins BCLAF1 and THRAP3, fragile X mental retardation 1 (FXR1) and its autosomal paralogues, fragile X mental retardation syndrome-related protein 1 and 2 (FXR1 and FXR2) and enhancer of rudimentary homolog (ERH). These interactions suggest that WTAP may link the m6A writer complex with other RNA processing activities, such as splicing, RNA stability or other nuclear events.

Despite advances in understanding the role of WTAP in m6A deposition, it remains unclear whether WTAP actively regulates m6A site selection or simply provides a scaffold for the complex. Future studies using live-cell imaging or inducible relocalization tools could resolve if speckles are sites of active m6A deposition.

### VIRMA: Directing methylation to the 3′ end of mRNAs

VIRMA is the largest component of the writer complex [55]. The VIRMA homolog in Drosophila, VIR, has an N-terminal domain which shares similarities with RNA and DNA helicases, as well as with ribonucleoproteins, indicating its potential involvement in RNA binding and processing [57]. However, direct RNA binding by this region has not yet been experimentally confirmed. Moreover, it is unclear whether the N-terminal domain contributes to RNA binding directly, functions in RNA remodeling or serves primarily as a structural scaffold. By contrast, electrophoretic mobility shift assays have shown that the C-terminal domain of VIRMA directly binds RNA. Truncation of the C-terminal 226 amino acids (resulting in VIRMA 1-1,586) produces a significant decrease in RNA binding affinity and almost complete loss of methylation activity [55]. These findings suggest that both the N- and C-terminal regions of VIRMA are crucial for effective RNA binding and for the catalytic function of the m6A writer complex [55]. However, it is not known whether VIRMA binds in a sequence-specific manner or if it non-specifically binds RNA. The significance of this RNA binding is also unclear, as the METTL3-METTL14 complex also binds RNA and specifically recognizes the DRACH site for methylation [14]. VIRMA may provide additional binding affinity or specificity for RNA by binding sites that are distant from the DRACH site, potentially supporting substrate positioning or complex stability.

Rather than being required for all m6A deposition, VIRMA is reported to mediate methylation near the terminal exon-exon junction [30]. By contrast, VIR is required for virtually all m6A deposition [20]. It remains possible that the study of mammalian VIRMA may have underestimated the importance of VIRMA, as knockdown was used. However, if VIRMA indeed mediates methylation near the terminal exon-exon junction, then VIRMA may selectively bind to this exon junction and recruit the m6A writer complex to this region. Proteomic studies have revealed that VIRMA interacts with polyadenylation cleavage factors such as CPSF5 and CPSF6, which are responsible for recognizing and cleaving the pre-mRNA at polyadenylation sites in the 3′ UTR [41]. This interaction suggests a mechanism by which VIRMA is directly recruited to mRNA and may simultaneously coordinate m6A deposition and transcription termination.

### RBM15: RNA binding and active targeting

Knockdown studies in mammalian cells [21] and genetic knockout studies in Drosophila [19] have shown that loss of RBM15 leads to near complete loss of m6A, indicating that RBM15 is crucial for effective m6A deposition. RBM15 has been extensively studied in pediatric leukemia, where chromosomal translocations of RBM15 are implicated in disease progression [58,59]. RBM15 and its paralog, RBM15B, which may also contribute to m6A modification [21], have also been linked to pre-mRNA splicing. Given that m6A deposition is strongly influenced by exon-intron architecture [30], RBM15’s connection to splicing is particularly intriguing. RBM15 appears to interact with the splicing machinery [60,61], potentially coupling splicing and methylation, and linking these processes to exonic methylation.

An alternative function of RBM15 may be to target the m6A writer complex to specific sites in mRNA and thus make nearby DRACH sites more susceptible to methylation. RBM15 contains three RNA recognition motifs (RRMs), which enable it to bind to RNA. RBM15 preferentially binds to U-rich sequences near m6A consensus sites, facilitating m6A methylation at these positions [21], and m6A peaks significantly overlap with RBM15 RNA binding sites [37]. Thus, like VIRMA, the RNA-binding functions of RBM15 may enhance m6A writer complex recruitment to RNA and add additional specificity to the methylation patterns in the transcriptome.

RBM15 also has a crucial role in nuclear export of RNAs, in part by binding to RNA and nuclear export factors [46,62]. In the case of retrotransposon RNAs, RBM15 mediates nuclear export by binding to specific sequence elements [62]. Thus, RBM15 may couple m6A deposition to nuclear export.

RBM15 may also be incorporated into the m6A writer complex on the basis of a phospho-dependent switch. RBM15 and RBM15B contain a Spen paralog and ortholog C-terminal (SPOC) domain, a conserved phosphoserine-binding domain that is found in several proteins [63]. The SPOC domain binds to a phosphorylated domain within WTAP and can pull down WTAP from cell lysates. These results suggest the possibility of dynamic phosphorylation-dependent assembly of the m6A writer complex [63]. Thus, pathways that promote phosphorylation of WTAP may also lead to the addition of RBM15 into the m6A writer complex and activation of methylation.

Furthermore, RBM15 protein levels may also be regulated. Recent studies have shown that RBM15 undergoes arginine methylation by PRMT1, making it a substrate for ubiquitination and degradation [47]. In this way, PRMT1 can markedly deplete RBM15 levels, which would be expected to reduce m6A levels. Because PRMT1 is activated by diverse stimuli, including ROS and calcium signaling pathways [64], many of these pathways may lead to altered RBM15 levels and altered m6A levels. Thus, RBM15, and possibly other subunits, may undergo degradation or regulated assembly into the m6A writer complex to regulate m6A levels.

Overall, the interaction domains in RBM15 make it likely to have a recruitment role in the m6A writer complex. However, whether RBM15 functions as an adapter that recruits the m6A writer complex to splice sites or to RNA Pol II or some other location, and the importance of these localizations, remains to be established.

### ZC3H13: Activation of the m6A writer complex

ZC3H13 is the least characterized component of the m6A writer complex, but it seems to stabilize VIRMA and have an activation function in the m6A writer complex. The cryo-EM structure shows extensive interactions between VIRMA and ZC3H13 [55]. Unlike WTAP and VIRMA, ZC3H13 lacks a rigid structure, with its C-terminal region wrapped around multiple domains of VIRMA. Thus, ZC3H13 may have an essential role in maintaining both the architecture and function of the complex.

By contrast, the interaction between ZC3H13 and WTAP is significantly weaker. The cryo-EM structure shows a CCCH-type zinc finger domain of unknown function at the ZC3H13 N-terminus, and an interaction domain with WTAP at the ZC3H13 C-terminus (amino acids 1,107 -1,669) [55]. Consistent with the cryo-EM findings, a study of the Drosophila ZC3H13 homolog, Flacc, indicates that Flacc is necessary for interaction of the Drosophila homologs of WTAP (Fl(2)d) and RBM15 (Nito), suggesting that ZC3H13 connects these key proteins within the m6A writer complex [20].

ZC3H13 is also essential for the activity of the m6A writer complex in vitro. Various m6A writer complex proteins were purified and used to reconstitute component parts of the complex, including the METTL3-METTL14 heterodimer and other complexes containing HAKAI, WTAP, VIRMA and ZC3H13 (C-terminus 1,106-1,668) in different combinations [55]. These studies showed that HAKAI was dispensable for methylation in vitro, and that VIRMA and WTAP, added together, were insufficient to induce activity of the METTL3-METTL14 heterodimer. However, the addition of ZC3H13 to VIRMA and WTAP lead to activation of methylation activity. Notably, the cryo-EM structures with and without ZC3H13 showed substantial rearrangement of the complex upon addition of ZC3H13. Although METTL3-METTL14 was not in the cryo-EM structure, the conformational change in VIRMA-WTAP-HAKAI complexes containing and lacking ZC3H13 raises the possibility that ZC3H13 could trigger an allosteric activation of METTL3-METTL14.

Although ZC3H13 is required for in vitro activity, its role in vivo in cells remains poorly understood. One possibility is that ZC3H13 functions as a molecular switch that toggles the m6A writer complex between active and inactive conformations.

### HAKAI: Potential roles in complex regulation and turnover

HAKAI is another auxiliary protein whose specific functions remain poorly characterized. HAKAI is a conserved component of the m6A methyltransferase complex across plants, Drosophila and human cells [25]. Its depletion in plants [24], flies and human cells [25] leads to a significant reduction in m6A levels. In Drosophila, loss of HAKAI disrupts m6A-dependent processes, including sex determination, while in human cells, HAKAI depletion destabilizes key subunits of the methyltransferase complex, leading to impaired m6A deposition [25].

HAKAI was first discovered as a cytosolic ubiquitin ligase for E-cadherin, a cell-cell adhesion protein [22]. Due to its ubiquitin ligase function, the presumptive function for HAKAI would be to ubiquitinylate and possibly degrade a nuclear target. The ubiquitination domain of HAKAI is essential for its dimerization and interaction with other m6A machinery members [25]. However, catalytically inactive mutants of HAKAI can still support m6A methylation by stabilizing the methyltransferase complex, suggesting that HAKAI’s ubiquitin ligase activity is dispensable for m6A methylation [25]. Thus, HAKAI’s function in the m6A writer complex seems to be independent of its ubiquitination function.

Given that HAKAI needs to be in the nucleus to be incorporated into the m6A writer complex, pathways that control its nuclear localization could lead to increased m6A formation. In Drosophila, different spliced isoforms of *Hakai* preferentially localize in either the nucleus or cytoplasm [25]. Studies in mammalian cells also show that HAKAI localization is dynamic and could be influenced by growth factors [65]. As with ZC3H13 and WTAP, subcellular localization of HAKAI could therefore be a regulatory mechanism for m6A deposition.

The reason why a protein with canonical E3 ligase activity, such as HAKAI, is incorporated into the m6A writer complex remains unknown. One possibility is that HAKAI serves as a regulatory factor that senses cellular stress and modulates the turnover or assembly of the complex. However, this hypothesis remains to be experimentally tested.

### Methylation-independent functions of m6A writer complex components

Although known for their role in catalyzing m6A modification, several components of the m6A writer complex also perform methylation-independent functions. For example, METTL3 promotes translation by directly binding the 3′ UTRs of target mRNAs and interacting with the translation initiation machinery, independent of its catalytic activity [66]. WTAP was originally characterized as a splicing regulator and continues to influence alternative splicing and RNA stability in ways not fully explained by m6A [67]. VIRMA interacts with polyadenylation factors and may help couple 3′ end processing with transcript fate independently of methylation [41]. HAKAI, known for its E3 ligase activity, may regulate mRNA export or protein turnover in contexts where its ubiquitin ligase function dominates [22,65]. These additional roles underscore that m6A writer complex components may have a broader regulatory impact beyond m6A deposition and warrant further study as multifunctional RNA-binding proteins.

## How might the regulation of auxiliary proteins regulate the m6A code?

In the previous sections, we have discussed how the auxiliary proteins could regulate the m6A writer complex. However, since the auxiliary proteins themselves are needed for m6A formation, their regulation could ultimately impact m6A levels. In this section, we discuss examples and mechanisms of regulation of various auxiliary proteins.

Although METTL3 is the catalytic component of the m6A writer complex [9], several early studies found that deletion of METTL3 only slightly reduced m6A levels [68–71]. This suggested that other methyltransferases accounted for considerable amounts of m6A in the cell. The discrepancies are now understood to stem largely from technical limitations. For example, residual m6A levels in many CRISPR-based METTL3 knock out experiments seem to derive from alternatively spliced but active METTL3 isoforms that appear after CRISPR-mediated indels are introduced into METTL3 [72]. Incomplete depletion via RNAi or partial gene editing may also explain residual methylation. More recent studies using knockout strategies or near-complete knockdown (>95%) confirm that METTL3 is responsible for more than 99% of m6A deposition in mRNA [72,73]. Therefore, the initial observation that METTL3 is dispensable for m6A deposition was due to methodological artifacts rather than redundancy of METTL3 in the m6A writer complex.

**Fig 3:**
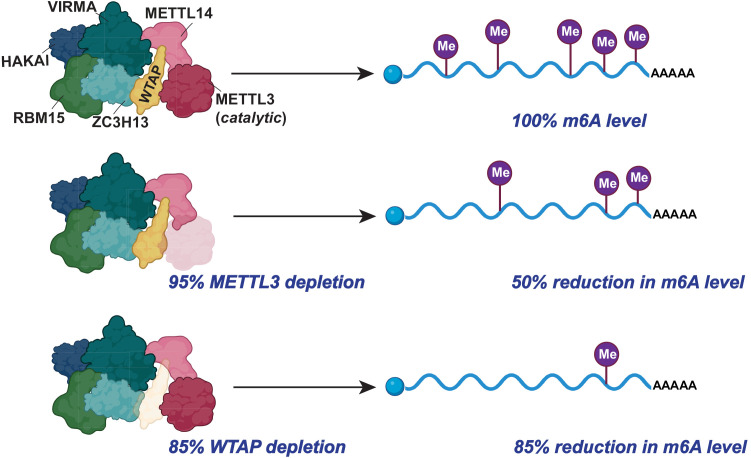
Auxiliary proteins may act as rate-limiting factors of the m6A writer complex. Here we use the term ‘rate-limiting’ to describe the functionally limiting component, based on knockdown correlations rather than direct kinetic measurements. About 50% of m6A remained when METTL3 was reduced to 5%, showing that only a small amount of METTL3 is required to maintain physiological levels of m6A in mRNA. This suggests that METTL3 is not the rate-limiting factor for m6A methylation. By contrast, reducing the level of the auxiliary protein WTAP (to 15%) caused a proportional decrease in m6A (to 15%), indicating a rate-limiting role of WTAP in m6A deposition. The abundance of each protein and its dynamic assembly into the m6A writer complex may regulate sensitivity to METTL3 depletion. Further work is needed to identify the relationships between METTL3 and other auxiliary proteins. Created in BioRender. Jaffrey, S. (2025) https://BioRender.com/alkhfzt.

Although required for methylation, the level of METTL3 may not determine the level of m6A, suggesting that, at least in some cell types, METTL3 might not be the rate-limiting subunit in the m6A writer complex. While the term rate-limiting typically refers to the kinetically limiting step in a biochemical reaction, here we use it to describe the functionally limiting component for methylation. Typically, the expression level of the rate-limiting component should directly correlate with the m6A level. An early study showed that substantial reductions in METTL3 do not significantly affect m6A levels. m6A levels were only affected when METTL3 was depleted by >95% [18], suggesting that METTL3 levels are much higher within a cell than the amount needed to achieve physiological mRNA m6A levels. However, reductions in WTAP lead to a corresponding reduction of m6A in human lung adenocarcinoma cells [18] (Fig 3). Similar effects were seen with VIRMA depletion [18], although these decreases with knockdown of VIRMA were smaller than those observed upon WTAP knockdown. These findings suggest that these auxiliary proteins may act as key rate-limiting factors in the m6A writer complex, significantly influencing m6A methylation. Notably, rate-limiting proteins are often regulated, highlighting the possibility that m6A levels may involve control of WTAP and/or VIRMA functions or levels. However, it remains unknown whether this rate-limiting behavior varies across cell types or physiological states such as stress, developmental stage or signaling inputs. Future studies using partial knockdowns of each auxiliary protein will help to reveal which are rate-limiting in different cell contexts.

Several lines of evidence indicate that WTAP expression is regulated to control m6A methylation levels. *WTAP* mRNA is also one of a few transcripts that undergo m6A-dependent splicing [37]. In experiments using rapid chemical degradation of METTL3, only a few genes showed altered splicing, notably including WTAP and RBM15 [37]. This type of regulation is consistent with a homeostatic pathway in which reduced m6A, as a result of METTL3 inhibition, leads to increased WTAP, which would presumably restore m6A levels. The fact that WTAP levels are preferentially induced during times of m6A depletion suggest that WTAP is a limiting factor in m6A synthesis. This feedback loop may represent a homeostatic mechanism to buffer fluctuations in m6A levels. An open question is whether this auto-regulation is generalized.

WTAP also has an unusually structured 5′ UTR, which is usually associated with translation regulation. Recent studies have shown that mTORC1 activation enhances the translation of WTAP mRNA by inducing unfolding of its 5′ UTR through the RNA helicase eIF4A/4B [74]. This regulatory mechanism allows mTOR to fine-tune WTAP protein levels in response to cellular growth signals, nutrient availability or stress. As WTAP is a core scaffold protein and may also be a rate-limiting protein in the m6A writer complex, controlling its expression via mTOR signaling ensures that m6A deposition is modulated based on the cell’s needs. These observations raise the possibility that WTAP functions as a signaling hub, linking nutrient sensing or stress-responsive pathways to RNA methylation. They also prompt broader questions about WTAP’s role, specifically, why it is uniquely positioned to transduce external signals into dynamic changes in the epitranscriptome.

Overexpression of VIRMA has also been linked to elevated m6A levels in breast cancer, where it drives tumorigenesis by regulating the unfolded protein response through the methylation of non-coding RNAs such as *NEAT1* [75]. Beyond cancer, it remains to be determined whether VIRMA levels are regulated to control m6A levels in cells, and whether these effects are on total m6A levels or at specific sites, such as at the terminal exon–exon junction.

Together, these findings highlight a central mystery: is m6A deposition governed by a ‘core’ complex with fixed composition, or by a flexible, modular system that assembles differently in response to cellular cues. Understanding how auxiliary proteins are regulated via transcription, splicing, translation, degradation and post-translational modification may provide key insights into how the m6A code is dynamically tuned.

## Conclusions and future directions

Understanding the exact roles of the individual m6A writer complex proteins will require high-resolution structural studies, such as cryo-EM, of the writer complex, in the presence or absence of possible regulatory modifications. Cryo-EM may provide insights into the assembly and interactions within the m6A writer complex, revealing novel details about its dynamics. While structures of the WTAP-VIRMA-HAKAI-ZC3H13 complex have been described [14,55], the fully assembled complex consisting of all seven members remains elusive. The structure of the m6A writer complex associated with RNA Pol II or other chromatin elements would help to reveal how the auxiliary subunits orchestrate the formation of these complexes.

Live-cell imaging techniques could offer real-time observations of the m6A writer complex and potentially its assembly during transcription. Live imaging of the writer complex, m6A formation and nuclear markers, such as markers for speckles, would provide valuable information about the timing and localization of m6A deposition. Additionally, genome-wide CRISPR screens could identify new interaction partners or regulators that link the m6A writer complex to other pathways, contributing to a more comprehensive model of m6A deposition.

Given the transient nature of interactions between its multiple protein components, it is essential to understand how the formation of the active complex is regulated and what mechanisms control its stability. Recent studies suggest that post-translational modifications (PTMs), particularly phosphorylation, may have a pivotal role in modulating these interactions. Since core components of the m6A writer complex, including METTL3, WTAP, VIRMA and HAKAI, are known to undergo phosphorylation [50], it is plausible that phosphorylation acts as a molecular switch regulating the assembly, conformation or activity of the m6A writer complex. Phosphorylation could serve as a signal that either promotes or inhibits complex formation, depending on the cellular context. For example, phosphorylation could induce conformational changes in the m6A writer complex proteins or alter their interaction with RNA, thereby affecting the efficiency of m6A methylation. However, the specific phosphorylation sites and the kinases responsible for these modifications remain largely unexplored. Future research should focus on mapping these phosphorylation events and determining how they influence the stability, localization and activity of the m6A writer complex.

Another line of investigation relates to the role of RBM15, which contains a SPOC domain that is reported to bind phosphorylated proteins [63]. This suggests that RBM15 may have a regulatory role in the phosphorylation-dependent assembly of the m6A writer complex. By interacting with phosphorylated components, RBM15 could influence the timing or location of m6A deposition, providing an additional layer of regulation. This interaction may act as a checkpoint, ensuring thatq1 the m6A writer complex is correctly assembled and recruited to RNA substrates only when needed. Exploring how RBM15 and its SPOC domain modulate the phosphorylation status of the m6A writer complex proteins will be an important avenue for future research.

In addition to phosphorylation, METTL3 and METTL14 undergo other PTMs, such as methylation and acetylation, that can significantly impact m6A deposition [76,77]. METTL14 can be arginine methylated, which alters its interaction with METTL3 and affects site selectivity and m6A levels [76]. METTL3 can be acetylated by the histone acetyltransferase p300, which impairs its methyltransferase activity and reduces m6A levels [77]. These findings suggest that m6A methylation is tightly regulated by PTMs, positioning the m6A writer complex as a downstream effector of stress, growth and differentiation cues. An open question is whether combinatorial PTMs serve as a ‘regulatory code’ that determines writer activity, localization or RNA target specificity under different physiological conditions.

The localization of the m6A writer complex to nuclear speckles suggests that speckles have a central role in coordinating m6A deposition. However, it remains unclear if m6A methylation is restricted to nuclear speckles or whether it also occurs in other subnuclear domains. Future studies could explore the spatiotemporal dynamics of m6A writer complex localization and investigate how these dynamics influence m6A levels under varying physiological or stress conditions.

Finally, understanding how changes in the assembly and localization of the m6A writer complex contribute to disease, particularly cancer, will be crucial. Abnormal m6A deposition due to alterations in the expression or phosphorylation of writer complex components could result in widespread changes in gene expression, promoting oncogenesis. Investigating these pathways could identify novel therapeutic targets for cancers and other diseases where m6A methylation has a critical role.

Future research should prioritize elucidating the regulatory mechanisms, such as phosphorylation, that govern the dynamics of the assembly of the m6A writer complex. These assays should be coupled to quantitative m6A mapping to determine how writer complex assembly is linked to methylation. Live-cell imaging of complex assembly may be particularly useful, especially when coupled with acute perturbations, to understand the principles and determine m6A writer complex assembly and how it might be impaired in disease processes, such as cancer. Understanding these processes will provide deeper insights into the regulation of RNA methylation and how it regulates gene expression.

## Abbreviations

EJC: exon junction complex
CBL or HAKAI: Cbl proto-oncogene like 1
m6A: N^6^-methyladenosine
m5C: 5-methylcytosine
NLS: nuclear localization signal
PTM: post-translational modification
PUS: pseudouridine synthase
RBM15: RNA-binding protein 15
RNA Pol II: RNA polymerase II
VIRMA: vir-like m6A methyltransferase associated
WTAP: Wilms-tumor associated protein
ZC3H13: zinc finger CCCH domain-containing protein 13
